# The *CHRNA5–A3–B4* Gene Cluster and Smoking: From Discovery to Therapeutics

**DOI:** 10.1016/j.tins.2016.10.005

**Published:** 2016-12

**Authors:** Glenda Lassi, Amy E. Taylor, Nicholas J. Timpson, Paul J. Kenny, Robert J. Mather, Tim Eisen, Marcus R. Munafò

**Affiliations:** 1 UK Centre for Tobacco and Alcohol Studies, School of Experimental Psychology, University of Bristol, Bristol, UK; 2 Oncology Translational Medicine Unit, Early Clinical Development, AstraZeneca, Cambridge, UK; 3 MRC Integrative Epidemiology Unit, University of Bristol, Bristol, UK; 4 Department of Neuroscience and Experimental Therapeutics Institute, Icahn School of Medicine at Mount Sinai, New York, NY, USA; 5 Neuroscience iMED, AstraZeneca, Cambridge, MA, USA; 6 Department of Oncology, University of Cambridge, Cambridge, UK

**Keywords:** *CHRNA5–A3–B4*, smoking behaviour, phenotype definition, tobacco-related disorders, precision medicine

## Abstract

Genome-wide association studies (GWASs) have identified associations between the *CHRNA5–CHRNA3–CHRNB4* gene cluster and smoking heaviness and nicotine dependence. Studies in rodents have described the anatomical localisation and function of the nicotinic acetylcholine receptors (nAChRs) formed by the subunits encoded by this gene cluster. Further investigations that complemented these studies highlighted the variability of individuals’ smoking behaviours and their ability to adjust nicotine intake. GWASs of smoking-related health outcomes have also identified this signal in the *CHRNA5–CHRNA3–CHRNB4* gene cluster. This insight underpins approaches to strengthen causal inference in observational data. Combining genetic and mechanistic studies of nicotine dependence and smoking heaviness may reveal novel targets for medication development. Validated targets can inform genetic therapeutic interventions for smoking cessation and tobacco-related diseases.

## Trends

Genome-wide association studies (GWASs) have highlighted genetic variants in the *CHRNA5–CHRNA3–CHRB4* gene cluster associated with smoking heaviness and nicotine dependence as well as known smoking-related diseases such as chronic obstructive pulmonary disease (COPD) and lung cancer.

Animal studies have described the anatomy and function of the nicotinic acetylcholine receptor (nAChR) subunits encoded by the *CHRNA5–CHRNA3–CHRB4* gene cluster.

More in-depth phenotyping in humans is improving our understanding of how these variants contribute to smoking behaviour.

Use of this gene cluster in Mendelian randomisation analyses is enabling us to investigate the causal effects of tobacco use. The latest findings suggest that smoking heaviness could be a causal factor for schizophrenia.

Validated genetic targets emerging from GWASs that influence smoking behaviour may pave the way towards individually tailored smoking cessation treatments, ultimately reducing the burden of smoking-related diseases.

## Genetic Variants and Smoking Behaviours

**Figure 1:**
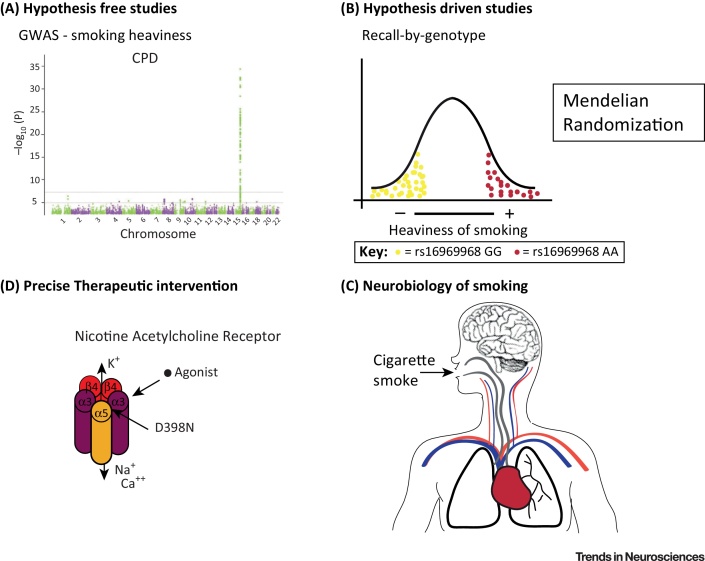
Key Figure: From Nicotine Consumption to Personalised Intervention through Genetic Studies

**GWASs** (see Glossary) seek to identify genetic variants associated with specific phenotypes, and cigarette smoking is a complex behavioural phenotype that has successfully been subjected to this approach (Figure 1A, Key Figure). An association between the SNP rs16969968 in ***CHRN**A5* and nicotine dependence was first reported in 2007 in a **candidate gene study** [1]. The following year rs1051730 at the same locus (in *CHRNA3* but strongly correlated with rs16969968 in samples of European ancestry) was found to be associated with smoking quantity in a GWAS [2]. This study also reported an association between rs1051730 and two smoking-related diseases: lung cancer and peripheral arterial disease. Importantly, *CHRNA5* was not recognised as a strong candidate gene at the time given the then-known neurobiology of tobacco dependence. Animal models had implicated the α4 and β2 nicotinic receptor subunits as critical to nicotine's reinforcing effects 3, 4, and α4β2* partial agonists are one of the most effective treatments available for smoking cessation [5]. Therefore, while the candidate gene study was published first, the GWAS (which did not require a strong prior hypothesis regarding gene selection) made the greater impact. What followed illustrates the potential for GWASs to advance our understanding of neurobiology in a way that hypothesis-driven investigations such as candidate gene studies (which rely on known or presumed neurobiology) rarely could. These findings have made variants in the *CHRNA5–CHRNA3–CHRNB4* region promising targets for the study of nicotine dependence and smoking heaviness, given their association with response to nicotine and its consequent consumption and titration.

## The CHRNA5–CHRNA3–CHRNB4 Gene Cluster

**Figure 2:**
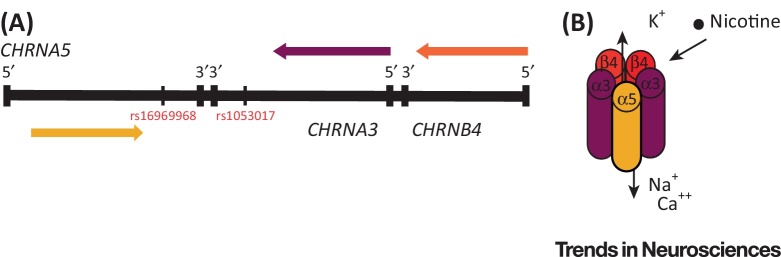
The Genetic Locus *CHRNA5–CHRNA3–CHRNB4* Encodes the Subunits of a Nicotinic Acetylcholine Receptor (nAChR). (A) Simplified illustration of the human *CHRNA5–CHRNA3–CHRNB4* gene cluster, which encodes the α3, α5, and β4 nAChR subunits (not to scale). *CHRNA5* is marked by rs16969968, which is in high linkage disequilibrium with rs1051730 in *CHRNA3*. (B) Subunit composition of the heteromeric α5–α3–β4 nAChR. Nicotine is an exogenous ligand for nAChRs; when nicotine binds to a nAChR, it modulates its trafficking of cations.

The *CHRNA5–CHRNA3–CHRNB4* gene cluster on chromosome 15 (at 15q25) encodes three (α3, α5, β4) of the eleven (α2–7, α9, α10, β2–4) neuronal nAChR (Figure 2A) subunits. The rs1051730 SNP in *CHRNA3* is a coding, synonymous variant that does not result in an **amino acid** change in the subsequent protein and is therefore unlikely to be of any functional significance; however, rs1051730 is highly correlated with rs16969968 in *CHRNA5*, a missense mutation that results in the substitution of aspartate (D) to asparagine (N) at the 398th amino acid in the resultant α5 subunit protein (D398N). This variant is functional; *in vitro* studies have demonstrated that α5 receptor complexes with the aspartic acid variant exhibit a twofold greater maximal response to a nicotine agonist compared with α5 receptor complexes containing the asparagine variant (i.e., the risk variant associated with smoking quantity and nicotine dependence [6]). Interestingly, an analysis of gene expression has shown that the risk allele of the rs1051730 SNP is associated with a lower level of expression of *CHRNA5* in the brain and peripheral blood mononuclear cells [7]. Animal studies have described the role of the α5 nAChR subunit by investigating the α5 **knockout-mouse model**'s phenotype; unfortunately, investigation of the role of the α3 subunit in nicotine intake is challenged by the early postnatal lethality that results from its genetic ablation.

## From GWAS to Neurobiology

Smoke inhaled from a cigarette is a mixture of chemicals that travel through the airways to the lungs. Nicotine is the primary addictive constituent of cigarette smoke; it is absorbed by the alveolar epithelium and then circulates in the bloodstream. Nicotine is an exogenous ligand for nAChRs and binds, after crossing the blood–brain barrier, to nAChRs ubiquitously expressed in the brain (Figure 1C). nAChRs are ligand-gated ion channels that open in response to the binding of acetylcholine and nicotine allowing trafficking of cations (Ca^++^, Na^+^, and K^+^; Figure 2B). Critically, our understanding of the neurobiology of smoking behaviours has been advanced by GWASs, particularly through the identification of how genetic variants that result in functional changes in nAChRs lead in turn to behavioural outcomes.

Following the identification of the association of the *CHRNA5–CHRNA3–CHRNB4* gene cluster with heaviness of smoking, an elegant series of experiments [8] established the underlying mechanism linking this genetic variation to nicotine response. This work utilised an α5 knockout-mouse model, which is analogous to individuals with reduced α5 receptor function (i.e., carriers of the rs16969968 risk allele). Both wild-type and knockout animals showed the expected inverted U-shaped dose–response curve for intravenous nicotine infusions with the difference that knockouts responded more vigorously at high doses. While wild-type mice appeared to titrate the delivery of nicotine dose (through self-administration) to achieve a consistent, desired level, knockout mice did so to a considerably lesser extent, consuming greater amounts as the dose increased.

Furthermore, [8] also showed that this effect could be ‘rescued’ in α5 knockout mice through the injection of a lentivirus vector into the medial habenula (MHb), rescuing expression of α5 subunits in this region. The knockout mice did not appear to differ from wild-type mice in their experience of the rewarding effects of nicotine, but the inhibitory effect of high nicotine doses on the activity of reward circuitries observed in wild-type mice appeared to have been largely abolished in knockout mice. This observation aligns with a previous study [9] where the differential effects of nicotine dose on reward between α5 knockouts and wild-types was illustrated using a conditioned place-preference task. In addition, a recent study in humans [10] found an attenuated aversive response to intravenously administrated nicotine in overnight-abstinent smokers who were carriers of the *CHRNA5* rs16969968 risk allele genotype. The MHb mainly projects to the interpeduncular nucleus (IPN) and [8] observed that diminished IPN activity in response to nicotine was seen in knockout mice. In short, it appears that high doses of nicotine stimulate the MHb–IPN tract through nAChRs containing α5 subunits and elicit aversion, limiting further intake. This does not occur when α5 signalling is deficient and consequently the negative effects of nicotine are attenuated. Similarly, smokers carrying the rs16969968 risk allele are therefore more likely to smoke more heavily than their counterparts without the risk allele.

The study reported in [11] provides further evidence that the MHb acts as a gatekeeper for nicotine intake. The authors manipulated the concentration of α5 and β4 *in vitro* while α3 was kept constant, and as a result the cation conductance of the channel was altered. nAChR activity changes according to the local electrostatic charge; therefore, the next step was to test the nicotine-evoked currents in MHb neurons of wild-type mice and transgenic mice (called Tabac mice) characterised by overexpression of β4. The authors [11] made patch-clamp recordings after local delivery of nicotine and reported a dramatically higher firing rate in Tabac mouse neurons. The behavioural response observed *in vivo* also showed significant changes in Tabac mice compared with wild-type mice. Tabac mice exhibited reduced nicotine intake and a strong preference for water rather than low-nicotine-concentration solutions in a two-bottle choice test. By contrast, their littermate controls drank water and nicotine solution equally even at much higher nicotine concentrations. Similarly, wild-type mice showed no preference for a nicotine-conditioned versus saline-conditioned chamber while Tabac mice spent less time in the latter. In addition, expression of the α5 D397N variant (corresponding to the human α5 variant D398N) was elicited in MHb neurons by injecting a lentivirus vector in Tabac mice. The latter restored their nicotine consumption and their two-bottle choice behaviour to a level comparable with wild types. In summary, α5 and β4 compete in regulating nicotine intake: β4 overexpression enhances MHb activity leading to aversion whereas induced α5 expression in Tabac mice normalised nicotine consumption.

## Measuring Smoking Behaviour

As we have seen, the first major results of GWASs of smoking behaviours related to nicotine dependence and smoking heaviness. These behaviours are conventionally quantified using self-report measures such as the Fagerström Test for Nicotine Dependence (FTND) and the number of cigarettes smoked daily (Box 1). Both the FTND and the number of cigarettes per day (CPD) are valuable measures, but since they are based on self-reporting they are prone to subjective errors. In addition, individuals differ in how they smoke a cigarette, varying in the number of puffs taken and the volume of smoke inhaled per puff and per cigarette (**smoking topography** [12]); therefore, cigarette smokers who consume the same CPD may differ in how much nicotine (and other toxicants) they consume. Individuals **homozygous** for the major allele at rs16969968 reduce the volume of smoke inhaled per puff when the nicotine content of the cigarette is increased; by contrast, carriers of the risk variant do not [13]. The lack of compensatory behaviour in risk allele carriers when smoking cigarettes with higher levels of nicotine is analogous to the reduced aversive effect of nicotine observed in α5 knockout mice [8].

How and what is measured to capture a phenotype (particularly for complex behaviours such as smoking heaviness) can be critical to the strength of the genetic association observed. Smoking studies that have used cotinine levels as a phenotype illustrate this. Nicotine consumed by a smoker is metabolised principally into cotinine, which is therefore a biomarker for nicotine consumption. It can be reliably detected in the serum, urine, and saliva of smokers (Box 1) [14]. In the study reported in [15], the authors showed that the variants in the *CHRNA5–A3–B4* gene cluster are more strongly associated with levels of cotinine than CPD. This was subsequently confirmed in an extensive meta-analysis: there is a much stronger signal for the association between the *CHRNA5–A3–B4* genotype and heaviness of smoking when this is captured by cotinine levels (4% of phenotypic variation explained) rather than CPD (1% variation explained). Interestingly, the association with cotinine levels remains when CPD is statistically adjusted for in the analysis 15, 16.

These findings highlight the need for more precise phenotypic measures. However, collecting detailed phenotypic data depletes resources and may not be feasible for large cohorts of individuals. A strategy to select a smaller sample is recall by genotype. For example, in 200 individuals of European ancestry from the general population, we would expect to find only 32 homozygous for the risk variant rs16969968 (AA, asparagine/asparagine) and 78 homozygous for the wild-type variant (GG, aspartate/aspartate) [17]. If in-depth phenotyping is time consuming and expensive, and collecting data on 200 participants is at the limit of available resources, selectively recruiting 100 minor homozygotes and 100 major homozygotes from a larger genetic screen before conducting in-depth phenotyping is likely to be a far more powerful and efficient use of resources, given the low cost of genotyping. We know that current smokers smoke one more cigarette and show a 138 nmol/l mean increase in cotinine levels for each copy of the rs16969968 minor allele that they may carry [16]. By recalling participants according to rs16969968 genotype (i.e., the two homozygote groups), we can capture the greatest phenotypic difference in smoking heaviness resulting from genetic difference at the *CHRNA5–A3–B4* locus (Figure 1B). As we see in the next section, this approach captures many of the features of a randomised experimental design and therefore any potential confounding factors should be randomly distributed across the sample because it has been selected solely according to genotype.

## Causal Analyses and Mendelian Randomisation (MR)

The robust association of variants in the *CHRNA5–A3–B4* gene cluster with smoking intensity phenotypes makes variation at this locus a valuable tool for investigating the causal effects of tobacco exposure. Although many of the harmful effects of smoking, such as lung cancer and cardiovascular disease, are well documented (http://www.surgeongeneral.gov/library/reports/50-years-of-progress/full-report.pdf), tobacco use is associated with many other conditions for which causal links remain to be established. For example, in the UK smoking rates are higher in individuals with a mental health condition – estimated to be 33% compared with 19% in the general population (http://www.ash.org.uk/files/documents/ASH_120.pdf). In addition, the mechanisms through which smoking causes some diseases, such as cardiovascular disease, are not yet fully understood. Much of the evidence regarding the health effects of smoking comes from observational data. Causal inference from observational data can be problematic because smoking is associated with a range of lifestyle and demographic factors including socioeconomic status, diet, and other substance use. Therefore, we cannot be certain that associations are due to smoking itself or to these other factors. For example, maternal smoking during pregnancy is linked with numerous offspring outcomes but confounding by familial factors (including genetic factors) is likely to explain some of these associations [18]. Furthermore, individuals may alter their smoking behaviour in response to ill health, so reverse causality may also influence these observational associations.

The problems of confounding and reverse causality in the investigation of the causal effects of smoking can be reduced by using variants in the *CHRNA5–A3–B4* gene cluster as proxies for smoking heaviness in MR analyses (Box 2). In contrast to direct measurement, germline genotypes reliably associated with risk factors can act as proxy measures of exposure, offering several advantages: genotypes are relatively easy to measure precisely, are stable through time, are largely immutable, and are not correlated with confounding factors, given the mechanisms of Mendelian inheritance 19, 20.

The study reported in [21] provides a useful example of how rs16969968 can be used in an MR approach. The authors found that the smoking heaviness-increasing allele of rs16969968 was associated with lower offspring birthweight in women who smoked during pregnancy but not in women who did not smoke during pregnancy. This confirmed previous findings that smoking in pregnancy is linked to reduced offspring birthweight and confirmed that this association is likely to be causal. In a similar manner, [22] demonstrates that the smoking heaviness-increasing allele of rs16969968 is associated with lower body mass index in current and **former smokers** but not in **never-smokers**, suggesting that heavier smoking causally lowers body weight. The finding has been replicated and extended to other measures of adiposity (e.g., waist circumference) in subsequent MR analyses 23, 24, 25. Similar analyses have shown that smoking leads to higher resting heart rate and may adversely affect kidney function, as it leads to higher estimated glomerular filtration rate 25, 26, as well as all-cause mortality [27].

MR has also highlighted observational associations that may not be causal. Despite strong links between smoking and mental health, MR studies have not found evidence that the minor allele of rs16969968 is associated with higher levels of depression in smokers 28, 29, 30, suggesting that smoking does not cause depression. Lack of associations of rs16969968 with blood pressure, serum lipids, and glucose levels indicate that smoking may not causally influence these cardiometabolic traits 25, 26.

Importantly, evidence for causal effects of smoking does not have to come from formally conducted MR studies. As discussed above, variants in the *CHRNA5–A3–B4* gene cluster were identified in GWASs of smoking-related diseases [e.g., lung cancer, chronic obstructive pulmonary disease (COPD)]. While there was debate about a possible direct effect of variants in the cluster on these diseases, it is now widely accepted that these variants appeared in these GWASs because these diseases are caused by smoking. GWASs can therefore provide us with important insights into whether environmental exposures may affect disease [31].

Recently, a GWAS of schizophrenia led to renewed interest in the relationship between smoking and schizophrenia. Smoking rates are high among individuals diagnosed with schizophrenia but it is unclear whether this link is causal and, if so, in which direction this operates. The identification of the *CHRNA5–A3–B4* cluster in the recent GWAS of schizophrenia [32] suggests either that variants in this gene cluster cause both schizophrenia and heavier smoking independently or that heavier smoking is a causal risk factor in the development of schizophrenia 33, 34, 35. As the GWAS was not conducted stratified by smoking status, it is difficult to distinguish between these two possibilities; however, a recent MR analysis [29], which was stratified by smoking status, provides supportive evidence for a causal effect of smoking on schizophrenia. They found that the minor allele of rs1051730 was associated with higher odds of being prescribed antipsychotic medication in **ever-smokers** but not in never-smokers in the Copenhagen General Population Study. A similar trend was observed for schizophrenia diagnosis. Other observational studies and meta-analyses have provided convergent evidence for the association between schizophrenia (and psychotic illness more generally) and smoking behaviour 36, 37, 38.

One limitation of the MR approach is that it typically requires very large sample sizes, because genetic variants for common traits generally only explain a very small proportion of phenotypic variation. Therefore, many MR studies combine data from multiple studies to increase their power to detect effects (e.g., [28]). A further solution is to perform MR analysis on samples of individuals recalled and phenotyped on the basis of genotype. As discussed above, this maximises genetic differences (and therefore differences in phenotypic exposure) between individuals. In the case of *CHRNA5–A3–B4*, selecting only homozygotes for rs16969968 increases the power to detect causal effects of smoking [17]. Investigating the causal effects of the genotype–phenotype relationship is key to progress in the identification of human genetic targets for smoking treatment.

## From Genetics to Therapeutics

The treatment of nicotine dependence remains a significant unmet medical need given the limited efficacy of behavioural and pharmacological [e.g., nicotine replacement therapy (NRT), varenicline] treatments. Most clinical studies of new chemical entities that may act as smoking cessation agents undertaken since 2012 have not progressed to the clinic due to lack of safety and/or efficacy [39]. At present in the USA there are only two new molecular agents, besides variations on NRT formulations, that are in clinical efficacy studies for smoking cessation. The first is a Phase II study looking at the efficacy of AZD8529, an mGluR2 **positive allosteric modulator**, being conducted by the National Institutes of Health (NIH)/National institute on Drug Abuse (NIDA) [NIDA (2016) The study of AZD8529 for smoking cessation in female smokers, ClinicalTrials.gov]. The second is a study directed by Cerecor, Inc. testing a kappa opioid receptor antagonist, CERC-501. Consistent with previous clinical trials, both of these mechanisms were identified and validated in preclinical rodent and primate intravenous nicotine self-administration models. This reflects our current reliance on preclinical models of aspects of nicotine dependence and their ability to identify and differentiate new mechanisms relevant in a manner that translates into clinical efficacy in human smokers.

Human genetics findings in nicotine dependence and smoking heaviness may reveal novel targets for medication development or even highlight novel brain substrates within which such targets are expressed. However, neither of the studies described above appears to have utilised human genetics to identify the target or to set inclusion criteria and thereby stratify patient populations to identify those most likely to demonstrate a positive response to treatment 40, 41. Nevertheless, it is clear that, relative to other substance use disorders (and more broadly across domains of psychiatry), human genetics approaches such as GWASs have proved successful in identifying allelic variations that influence vulnerability to nicotine dependence. There is emerging evidence that a stratified medicine approach utilising *CHRNA5* genetic biomarkers may improve patient responsiveness to NRT 10, 42, 43, 44. For example, [42] describes two randomised cessation trails in which it was found that the efficacy of NRT varied with rs16969968 genotype while the efficacy of varenicline did not, as subjects responded to varenicline regardless of CHRNA5 rs16969968 genotype.

The α5*-containing nAChRs impacted by *CHRNA5* variation may represent an important target for medication development. Considering that *CHRNA5* risk alleles result in decreased function of α5* nAChRs incorporating the variant subunits 6, 45, 46, it is reasonable to predict that novel chemical entities that enhance activity of a5* nAChRs will decrease tobacco use 8, 10. Moreover, the neuronal circuits in which α5* nAChRs that regulate nicotine intake may reside, and other genes associated with risk of nicotine dependence, are also likely to contain novel targets for medication development. Studies in patients with the rs16969968 risk variant suggest that diminished activity of the α5-containing nAChR leads to a delay in response to NRT and smoking cessation 5, 11. Given the observed reduction in activity of the D398N α5 nAChR, it seems logical to begin the identification of selective ligands that enhance nAChR function (i.e., agonists; Figure 1D). However, the highly conserved nature of the orthosteric site among nicotinic acetylcholine receptor subunits makes it challenging to identify orthosteric ligands with optimal selectivity and physiochemical properties [47]. Therefore, an alternative approach is to look for selective positive allosteric modulators of the α5 subunit, perhaps by targeting key habenular circuits. This could be achieved either by restoring appropriate cholinergic tone and the aversive or reward-attenuating properties of nicotine in a genetically insensitive population or by increasing it in the normal population to reduce daily nicotine intake [5].

## Concluding Remarks and Future Perspectives

GWASs have highlighted the important role of the *CHRNA5–A3–B4* gene cluster in smoking heaviness. Further studies have highlighted that the same gene cluster is also associated with vulnerability to many smoking-associated diseases such as COPD and lung cancer 1, 2, 6, 48, 49. Whether the same genes are associated with mental health is still disputed, although the latest findings suggest that smoking heaviness and schizophrenia share some genetic liability 35, 38.

Early studies on the *CHRNA5–CHRNA3–CHRNB4* cluster and smoking behaviours relied on self-reported phenotypes (such as CPD) that display great variance, but subsequent studies have replicated the gene-to-phenotype associations with novel measures and analysis tools that allow a further step towards understanding the directionality in the relationship between genes and behaviour.

The advances in phenotyping (e.g., by employing precise biomarker assessments), together with the fast development of DNA and RNA sequencing technologies and powerful analytical tools, are making GWASs easier to perform and more informative. Further genomic mapping of smoking phenotypes will clarify the role of the *CHRNA5–A3–B4* gene cluster and, more broadly, of sets of variants and may lead to the development of **polygenic risk scores** for such complex traits and, ultimately, clarify the pathways that may be shared by multiple phenotypes. Genetic variants emerging from GWASs that influence smoking behaviour and tobacco-related diseases, and that are validated by independent studies, could inform individual-tailored treatments (see Outstanding Questions) and pave the way to targeted genetic therapeutic interventions for smoking cessation, ultimately reducing the burden of tobacco-related diseases.

Outstanding Questions Will human GWASs, in parallel with experiments in preclinical (e.g., rodent) models and human studies, allow us to go beyond the known neurobiology of smoking behaviour and provide genuinely novel mechanistic insights?

Will biomarkers (e.g., cotinine) allow us to both gain information about the role of target genes and anticipate the response to treatments targeting the corresponding gene products?

Will insights into gene-to-phenotype causal relationships lead to novel treatments for smoking cessation?

Do schizophrenia and heavy smoking share an underlying genetic liability or is smoking causally related to schizophrenia risk or other mental health conditions?

Will increasing the reward-attenuating properties of nicotine enhance treatment response in a well-defined population of smokers?

## Figures and Tables

**Figure 3:**
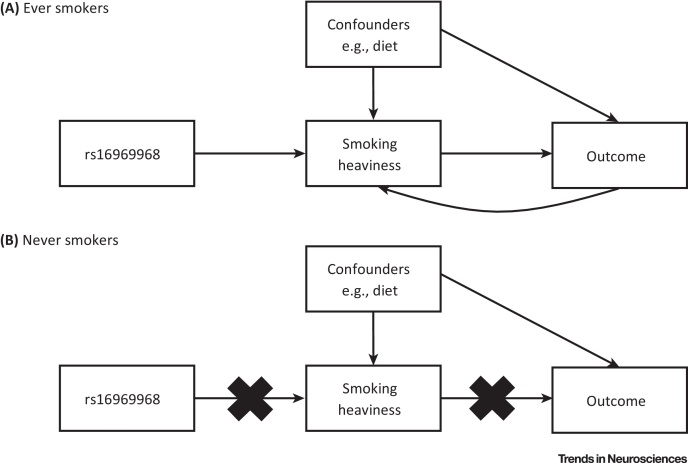
Mendelian Randomisation Using rs16969968 in *CHRNA5* as an Instrument for Smoking Heaviness. (A) In Mendelian randomisation analyses, rs16969968 is used as a proxy for smoking heaviness in ever-smokers. While the association between smoking heaviness (e.g., number of cigarettes smoked per day) and the outcome of interest may be confounded by lifestyle and demographic factors (e.g., diet), the association between rs16969968 and the outcome should not be. The outcome of interest may influence smoking heaviness (reverse causality) but will not influence rs16969968 genotype. (B) As never-smokers do not smoke, any association between genotype and outcome cannot be operating through effects of the genotype on smoking.

## Box 1 Phenotyping of Smoking Behaviours

Phenotyping in humans can rely on subjective methods. Precise phenotypes such as proximate biomarkers better capture genetically disposed interindividual differences in smoking. Here we provide a brief description of common indicators used to quantify smoking dependence and heaviness.

**CPD**

CPD is the count of cigarettes smoked in one day from waking up to going to bed. This phenotype has been used in several studies 2, 50, 51.

**Questionnaires**

• The FTND, or Fagerström Test for Cigarette Dependence (FTCD) as Fagerström renamed it in 2012 [52], assesses the intensity of physical addiction to nicotine. The FTND is a six-item questionnaire. The total score range is 0–10 and a higher score indicates higher dependence. Often, smokers with a score greater than 4 are defined as dependent 1, 6.
• The Questionnaire of Smoking Urges (QSU) provides a measure of current craving levels. The QSU is a ten-item questionnaire. Each item requires a 1–5 response and a higher score indicates a greater urge to smoke.

**Smoking Topography**

Objective indicators such as number of puffs, puff volume, and the duration and velocity of the smoking of a cigarette highlight interindividual differences in smoking behaviour. They can lead to different levels of biomarkers for similar levels of CPD.

**Biomarkers** [53]

Biomarkers are directly correlated with the level of smoking and are measured objectively.

• Carbon monoxide (CO): Expired air CO levels in the breath are measured via a portable CO analyser. Smoking status is usually confirmed by a value of 10 parts per million (ppm). CO testing is a rapid, low-cost, and noninvasive method for confirming smoking status [54]. Alternatively, carboxyhaemoglobin (COHb) can be measured spectrophotometrically in blood. Environmental sources of CO (e.g., car exhaust fumes) can contribute to the overall level of CO in the organism.
• Nicotine: Nicotine levels can be measured in urine, saliva, and blood by means of gas or high-performance liquid chromatography and immunoassays. However, nicotine has a short half-life and is therefore suited to capturing nicotine consumption only in the 8–12 h before testing.
• Cotinine: Most (∼90%) nicotine is broken down into cotinine. The half-life of cotinine is relatively long (∼16 h) and it is therefore generally preferred as a biomarker of nicotine intake. Urine tests allow discrimination between smokers and non-smokers while more accurate measures, in saliva and blood, are performed as for nicotine, by means of gas or high-performance liquid chromatography and immunoassays.

## Box 2 MR Analysis

MR analysis is a type of instrumental variable analysis that uses genetic variants (either individually or combined in a genetic risk score) as proxies for measured exposures (in this case smoking) in observational datasets (see Figure 3 in main text) [19]. As independent heritable units (be they specific variants, structural changes, or genetic risk scores) are inherited independently of the environment, they should not be associated with other factors that confound the relationship between exposures and outcome measures. In addition, germline genetic changes reflect life-course alterations and (while these effects may be variable through time) they will be less susceptible to reverse causality. To be a suitable instrument for a MR analysis, a genetic variant should be: (i) robustly associated with the exposure we are interested in; (ii) associated with the outcome measure only through the exposure of interest (i.e., it does not have **pleiotropic effects**); and (iii) not associated with factors that confound the association between exposure and outcome. It is important to note that new methods for the undertaking of MR analyses are able to account for violations of these assumptions and to quantify the extent of the likely biases induced by failures to satisfy them 55, 56.

While it is not possible to fully test these assumptions, knowledge of the biology of the *CHRNA5–CHRNA3–CHRNB4* gene cluster, including the identification of the functionally significant SNP rs16969968 and its role in determining response to nicotine, strengthens the validity of variants in this gene cluster as instruments for MR analyses.

The rs16969968 (or rs1051730) variant has been used to investigate whether associations between smoking and several health outcomes are causal. As the variant is associated with smoking heaviness rather than smoking initiation, it is used as an instrument for smoking heaviness within smokers and findings of MR studies using this variant do not directly tell us whether being a smoker causes a particular disease. However, using rs16969968 as an instrument for smoking heaviness in smokers provides evidence about whether smoking has a dose–response effect on an outcome (which is a key criterion for causality). MR analyses using this variant are stratified by smoking status (never- and ever-smokers or never-, former, and current smokers) and we test for an interaction between genotype and smoking status. As there are no effects of the variant on smoking heaviness in never-smokers, this group can be used to test the assumption of no pleiotropy. If an association with the outcome of interest is observed in never-smokers, it suggests that the variant is operating through a pathway other than smoking and that the MR assumptions may be violated.

## Glossary

Amino acid: the subunit that, joined together with other amino acids, forms a protein.
Candidate gene association study: a study in which a specific genetic variant is selected because of its perceived biological relevance to the phenotype of interest (i.e., hypothesis driven).
CHRN*: cholinergic receptor, nicotinic*.
Ever-smokers: individuals who either currently smoke or who used to smoke.
Former smokers: individuals who used to smoke but have stopped.
Genome-wide association study (GWAS): examines the associations of SNPs of the whole genome of a cohort of individuals with the phenotype of interest. The approach has no *a priori* hypotheses.
Haplotype: combination of multiple SNPs inherited together from a single parent.
Homozygous: individuals are homozygous when they have two of the same allele at a given locus.
Knockout-mouse models: mice in which a target gene has been inactivated.
Never-smokers: individuals who have never smoked
Pleiotropic effects: when a SNP or gene influences more than one trait.
Polygenic risk scores: individual scores reflecting the aggregate genetic burden from collections of common variants (which can be formed by a variety of methods, the simplest being summed allele counts).
Positive allosteric modulator: a ligand that binds to a receptor and modulates its activity.
Smoking topography: how a cigarette is smoked (i.e., the number of puffs taken and the volume of smoke inhaled per puff and per cigarette).

## Supplemental information

Answers to Interactive Questions
