# Supplementary material for: The CHRNA5–A3–B4 Gene Cluster and Smoking: From Discovery to Therapeutics
Source: Trends Neurosci. 2016 Dec;39(12):851–61. doi: 10.1016/j.tins.2016.10.005 (PMC5152594; doi:10.1016/j.tins.2016.10.005)
Supplement: Answers to Interactive Questions [file mmc1.pdf]

Question 1:

1. Which variants within the CHRNA5-CHRNA3-CHRNB4 region are promising targets for the study of nicotine dependence and smoking heaviness?

● rs16969968 and rs1051730

Explanation:

An association between the single nucleotide polymorphism (SNP) rs16969968 in CHRNA5 and nicotine dependence was first reported in 2007 in a candidate gene study. The following year, rs1051730 at the same locus (in CHRNA3, but strongly correlated with rs16969968 in samples of European ancestry) was found to be associated with smoking quantity in a GWAS.

Reference:

Saccone, S.F., et al., Cholinergic nicotinic receptor genes implicated in a nicotine dependence association study targeting 348 candidate genes with 3713 SNPs. Hum Mol Genet, 2007. 16(1): p. 36-49. Thorgeirsson, T.E., et al., A variant associated with nicotine dependence, lung cancer and peripheral arterial disease.

❍ rs1040208 and rs56869968

Question 2:

Both alpha5 knockout mice and wild-types show an inverted U-shaped dose-response curve for intravenous nicotine infusions. In what way do knockout mice differ from wild-type mice?

● The knockouts responded more vigorously at high doses

Explanation:

the inhibitory effect of high nicotine doses on the activity of reward circuitries observed in wild-type mice appeared to have been largely abolished in knockout mice.

Reference:

Fowler, C.D., et al., Habenular alpha5 nicotinic receptor subunit signalling controls nicotine intake. Nature, 2011. 471(7340): p. 597-601

❍ The knockouts did not work at all

Question 3:

Which of the followings is a biomarker for nicotine consumption?

● Cotinine

Explanation:

Nicotine consumed by a smoker is metabolised principally into cotinine

Reference:

Ware, J.J., et al., Genome-Wide Meta-Analysis of Cotinine Levels in Cigarette Smokers Identifies Locus at 4q13.2. Sci Rep, 2016. 6: p. 20092. Keskitalo, K., et al., Association of serum cotinine level with a cluster of three nicotinic acetylcholine receptor genes (CHRNA3/CHRNA5/CHRNB4) on chromosome 15. Hum Mol Gen

❍ Cigarettes smoked per day

Question 4:

How might you investigate whether associations between smoking and health outcomes are causal?

● Mendelian randomization

Explanation:

Mendelian randomization analysis is a type of instrumental variable analysis which uses genetic variants (either individually or combined in a genetic risk score) as proxies for

measured exposures (in this case smoking) in observational datasets.

Reference:

Smith, G.D. and S. Ebrahim, 'Mendelian randomization': can genetic epidemiology contribute to understanding environmental determinants of disease? International Journal of Epidemiology, 2003. 32(1): p. 1-22

❍ ANOVA
